# Molecular phylogeny of the *Drosophila obscura *species group, with emphasis on the Old World species

**DOI:** 10.1186/1471-2148-7-87

**Published:** 2007-06-07

**Authors:** Jian-jun Gao, Hide-aki Watabe, Tadashi Aotsuka, Jun-feng Pang, Ya-ping Zhang

**Affiliations:** 1 Laboratory for Conservation and Utilization of Bio-resources, Yunnan University, Kunming, Yunnan, China; 2 State Key Laboratory of Genetic Resource and Evolution, Kunming Institute of Zoology, Chinese Academy of Sciences, Kunming, Yunnan, China; 3 Biological Laboratory, Sapporo College, Hokkaido University of Education, Sapporo, Japan; 4 Department of Biology, Tokyo Metropolitan University, Japan

## Abstract

**Background:**

Species of the *Drosophila obscura *species group (e.g., *D. pseudoobscura*, *D. subobscura*) have served as favorable models in evolutionary studies since the 1930's. Despite numbers of studies conducted with varied types of data, the basal phylogeny in this group is still controversial, presumably owing to not only the hypothetical 'rapid radiation' history of this group, but also limited taxon sampling from the Old World (esp. the Oriental and Afrotropical regions). Here we reconstruct the phylogeny of this group by using sequence data from 6 loci of 21 species (including 16 Old World ones) covering all the 6 subgroups of this group, estimate the divergence times among lineages, and statistically test the 'rapid radiation' hypothesis.

**Results:**

Phylogenetic analyses indicate that each of the *subobscura*, *sinobscura*, *affinis*, and *pseudoobscura *subgroups is monophyletic. The *subobscura *and *microlabis *subgroups form the basal clade in the *obscura *group. Partial species of the *obscura *subgroup (the *D. ambigua*/*D. obscura*/*D. tristis *triad plus the *D. subsilvestris*/*D. dianensis *pair) forms a monophyletic group which appears to be most closely related to the *sinobscura *subgroup. The remaining basal relationships in the *obscura *group are not resolved by the present study. Divergence times on a ML tree based on mtDNA data are estimated with a calibration of 30–35 Mya for the divergence between the *obscura *and *melanogaster *groups. The result suggests that at least half of the current major lineages of the *obscura *group originated by the mid-Miocene time (~15 Mya), a time of the last developing and fragmentation of the temperate forest in North Hemisphere.

**Conclusion:**

The *obscura *group began to diversify rapidly before invading into the New World. The *subobscura *and *microlabis *subgroups form the basal clade in this group. The *obscura *subgroup is paraphyletic. Partial members of this subgroup (*D. ambigua*, *D. obscura*, *D. tristis*, *D. subsilvestris*, and *D. dianensis*) form a monophyletic group which appears to be most closely related to the *sinobscura *subgroup.

## Background

Species of the *Drosophila obscura *group (41 species assigned to six subgroups) are mostly inhabitants of temperate forest throughout the Holarctic region, with some can adapted into high-elevation temperate-like habitats in the Afrotropical, Neotropical and Oriental regions. Some of these species (e.g., *D. pseudoobscura *and its close relatives) have served as favorable models for evolutionary biology since the influential works of Dobzhansky and his colleagues in the 1930's [1,2]. The whole-genome sequence of *D. pseudoobscura *was determined following *D. melanogaster*. Comparisons between the two species have shed new light on *Drosophila *genome evolution [3]. In addition, in the past two decades, increasing number of evolutionary studies have been conducted in a historical background of the *obscura *species group on varied subjects, e.g., evolution of genome size [4], evolution of karyotype and *P *elements [5], origin and evolution of *Drosophila *Y chromosome [6] and genetics of morphological evolution [7].

Since the 1950's, a number of studies have been conducted to reconstruct phylogeny of the *obscura *group via a variety of approaches [2,8]. Recent molecular phylogenetic studies [9-13] clearly support the monophyly of the *obscura *species group and recover several well-supported lineages, for example, the *affinis*, *pseudoobscura*, and *subobscura *subgroups, the *D. ambigua *triad (*D. obscura*, *D. ambigua *and *D. tristis*), give essential support to the monophyletic origin of the New World species, i.e., those of the *affinis *and *pseudoobscura *subgroups. In spite of this, the relationship among the Old World *obscura*, *subobscura*, *microlabis*, and *sinobscura *subgroups, and their relationship to the New World clade are still poorly resolved. This phylogenetic predicament was partially ascribed to the "rapid radiation" history of the *obscura *group [10,12]. An alternative hypothesis to explain the lack of resolution at the base of this phylogeny is a bias in taxon sampling. For example, none of the previous phylogenetic studies has dealt with the *obscura *group as a whole: different studies employed different set of taxa, with species from the Afrotropical region (5 species) and Oriental region (8 described + 2 undescribed species) have rarely been investigated [14,15], probably due to the difficulty in collecting and/or culturing these poorly known taxa.

In the present study, nucleotide sequences (henceforth referred to as NT) from six loci (Table 1) of 21 *D. obscura *group species and 2 *D. melanogaster *group species (Table 2) are used to reconstruct the phylogeny of the *obscura *species group. Phylogenetic analyses are also performed based on translated amino acid sequences (henceforth referred to as AA). We then estimate the divergence times in the *obscura *group, and statistically test the previously proposed "rapid radiation" hypothesis [10,12]. Finally, the evolutionary history of the *obscura *group is discussed.

**Table 1:** Gene loci sampled in the present study. Numbers show aligned lengths and numbers of parsimony informative sites (PI, given in parentheses) for nucleotide or translated amino acid sequences of each locus.

Gene loci	Nucleotide sequences	Translated amino acid sequences
Mitochondrial genes		
NADH dehydrogenase subunit 2 (*ND2*)	1029 (292)	342 (69)
Cytochrome oxidase subunit I (*COI*)	496 (127)	158 (3)
Cytochrome oxidase subunit II (*COII*)	688 (171)	230 (16)
Cytochrome b (*Cyt b*)	893 (232)	296 (18)
Nuclear genes		
Alcohol dehydrogenase (*Adh*)	370 (84)	123 (14)
28S ribosomal RNA (*28S*)	650 (33)	-
Total	4126 (939)	1149 (120)

**Table 2:** Species sampled in the present study and collection data of samples used for DNA sequencing.

Species group	Species subgroup	Species	Geographical Origin
*obscura*	*affinis*	*affinis*	Pennsylvania, USA*
		*helvetica*	Unknown site, Switzerland
	*pseudoobscura*	*pseudoobscura*	California, USA*
		*persimilis*	Unknown site, USA*
		*miranda*	Unknown site, USA*
	*obscura*	*ambigua*	Vienna, Austria
		*obscura*	Tuebingen, Germany
		*tristis*	Tuebingen, Germany
		*subsilvestris*	Tuebingen, Germany
		*dianensis*	Kunming, Yunnan, China
		*bifasciata*	Yakutsuk, East Siberia, Russia
		*imaii*	Sapporo, Japan
		*limingi*	Kunming, Yunnan, China
		*tsukubaensis*	Koganei, Tokyo, Japan
	*sinobscura*	*sinobscura*	Chitou, Taiwan, China
		*luguensis*	Lugu Lake Nature Reserve, Yunnan, China
		*hubeiensis *(HB)	Shennongjia Nature Reserve, Hubei, China
		*hubeiensis *(KM)	Kunming, Yunnan, China
	*subobscura*	*subobscura*	Helsinki, Finland
		*guanche*	Canary Is.
		*madeirensis*	Madeira Is.
	*microlabis*	*microlabis*	Mt. Elgon, Kenya
*melanogaster*	*melanogaster*	*melanogaster*	All sequences from GenBank
		*yakuba*	All sequences from GenBank

* Stocks from the Bowling Green Stock Center, with missing stock numbers.

## Results

### Summary of the DNA sequences

The alignment of the six gene regions spanned 4,126 nucleotide or 1,149 amino acid positions (Table 1). Table 3 gives accession numbers for the nucleotide sequences, either cited from GenBank [9-11,13,15-28] or newly determined for this study.

**Table 3:** Accession numbers for sequences. Sequences with underlined accession numbers are used for the statistical test of temporal pattern only.

	*ND2*	*COI*	*COII*	*Cyt b*	*Adh*	*28S*
*D. luguensis*	EF216229	EF216247	EF216257	EF216273	EF216308	EF216295
*D. sinobscura*	EF216236	EF216249	EF216259	EF216280	U90954 [15]	EF216303
*D. hubeiensis *(HB)	EF216225	EF216243	EF216253	EF216269	U90953 [15]	EF216291
*D. hubeiensis *(KM)	EF216226	EF216244	EF216254	EF216270	EF216312	EF216292
*D. dianensis*	EF216222	EF216241	EF216251	EF216266	EF216310	EF216288
*D. subsilvestris*	EF216237	U51616 [11]	EF216260	EF216281	AF067283 [27]	EF216304
*D. bifasciata*	EF216221	U51611 [11]	M95147 [9]	EF216265	U40986 [20]	EF216287
*D. imaii*	EF216227	EF216245	EF216255	EF216271	U40987 [20]	EF216293
*D. tsukubaensis*	EF216239	EF216250	EF216261	EF216283	AF067284 [27]	EF216306
*D. limingi*	EF216228	EF216246	EF216256	EF216272	EF216309	EF216294
*D. obscura*	EF216233	U51614 [11]	AF081356 [13]	EF216277	U90955 [15]	EF216300
*D. ambigua*	EF216220	U51610 [11]	M95145 [9]	EF216264	X54813 [21]	EF216286
*D. tristis*	EF216240	U51617 [11]	EF216262	EF216284	U90956 [15]	EF216307
*D. subobscura*	EF216238	U51615 [11]	M95151 [9]	EF216282	M55545 [22]	EF216305
*D. madeirensis*	EF216230	U51613 [11]	AF081355 [13]	EF216274	X60112 [23]	EF216296
*D. guanche*	EF216223	U51612 [11]	AF081354 [13]	EF216267	X60113 [23]	EF216289
*D. affinis*	EF216219	U51604 [11]	M95140 [9]	EF216263	AF067280 [27]	EF216285
*D. algonquin*	-	-	M95144 [9]	U07279 [10]	-	-
*D. athabasca*	-	-	M95141 [9]	-	-	-
*D. azteca*	-	U51605 [11]	M95146 [9]	U07283 [10]	-	X71205 [17]
*D. narragansett*	-	-	M95149 [9]	-	-	-
*D. tolteca*	-	-	M95152 [9]	-	AF081357 [13]	-
*D. helvetica*	EF216224	EF216242	EF216252	EF216268	AF067282 [27]	EF216290
*D. pseudoobscura*	EF216235	U51602 [11]	M95150 [9]	EF216279	X68164 [24]	EF216302
*D. persimilis*	EF216234	U51609 [11]	M95143 [9]	EF216278	M60997 [25]	EF216301
*D. miranda*	EF216232	U51608 [11]	M95148 [9]	EF216276	M60998 [25]	EF216299
*D. lowei*	-	-	M95142 [9]	-	-	-
*D. microlabis*	EF216231	EF216248	EF216258	EF216275	EF216311	EF216298
*D. melanogaster*	NC_001709 [16]	U51619 [11]	J01404 [19]	NC001709 [16]	M17833 [26]	EF216297
*D. yakuba*	X03240 [17]	X03240 [18]	X03240 [18]	X03240 [18]	X54120*	X71167 [28]

* Ashburner, unpublished data.

**Figure 1:**
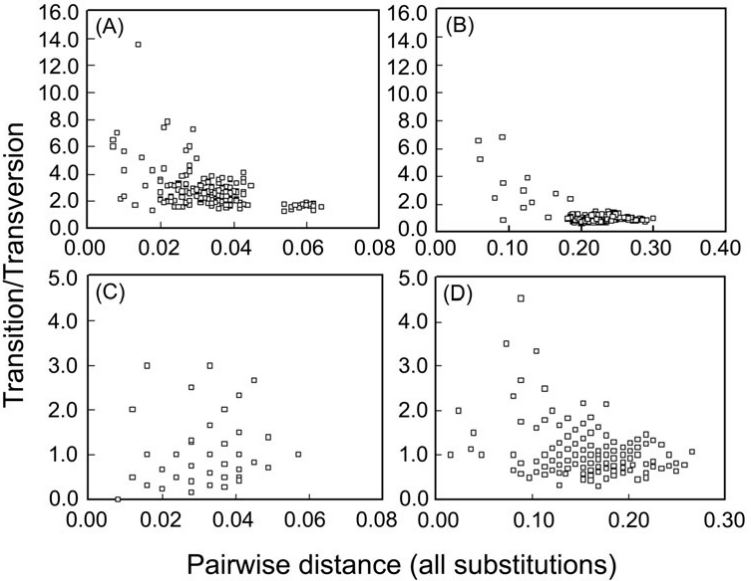
**Saturation plots for nucleotide substitutions**. For each comparison, ratios of ti/tv for pairs of sequences (Y-coordinate) are graphed versus the corresponding numbers of total substitutions (X-coordinate). (A) and (B), concatenated sequence of the mitochondrial genes, 1st+2nd and 3rd codon positions, respectively; (C) and (D), *Adh *sequence, 1st+2nd and 3rd codon positions, respectively.

A plot of nucleotide substitution saturation is shown in Figure 1. For the mitochondrial sequences, the transition/transversion (ti/tv) ratio in 1st+2nd codon positions (Figure 1A) ranges from 13.50 (*D. ambigua *vs. *D. obscura*) to 1.313 (*D. dianensis *vs. *D. guanche*), with the average of 2.70; and the ratio in the 3rd codon position (Figure 1B) ranges from 6.750 (*D. pseudoobscura *vs. *D. miranda*) to 0.581 (*D. affinis *vs. *D. madeirensis*), with the average of 1.08. This suggests strong saturation in the 3rd codon positions between distantly related species. For the *Adh *gene, the ti/tv ratio in 1st+2nd codon positions (Figure 1C) ranges from 3.00 (e.g., *D. ambigua *vs. *D. guanche*) to 0.00 (*D. miranda *vs. *D. persimilis*); the ratio in the 3rd codon positions ranges from 4.50 (*D. limingi *vs. *D. tsukubaensis*) to 0.429 (*D. obscura *vs. *D. affinis*/*D. limingi*). Slight decrease of ti/tv is found in the 3rd codon positions with increase of pairwise distance.

### Pairwise partition homogeneity test (PHT)

Table 4 shows the results of pairwise PHT between NT partitions. On a threshold of *P *= 0.05 [29], incongruences are found in 4 out of 15 pairwise tests (*ND2 *vs. *COI*, *ND2 *vs. *Adh*, *COI *vs. *COII*, and *COI *vs. *Adh*) with un-weighted parsimony scheme. Compared to this, when a six-parameter weighting scheme (see below for details) was implemented, the *P *values for most pairwise comparisons increased greatly.

**Table 4:** Results of pairwise partition homogeneity test (PHT). Numbers above and below diagonal show *P*-values resulted of the un-weighted and the six-parameter weighting methods, respectively.

	*ND2*	*COI*	*COII*	*Cyt b*	*Adh*	*28S*
*ND2*		0.026*	0.079	0.353	0.010*	0.925
*COI*	0.082		0.012*	0.083	0.005*	0.984
*COII*	0.346	0.135		0.354	0.142	0.855
*Cyt b*	0.196	0.118	0.365		0.149	0.996
*Adh*	0.187	0.076	0.297	0.202		0.892
*28S*	0.938	0.984	0.988	0.956	0.843	

Incongruence between data partitions indicates that the two partitions compared have had different histories or that one of them violate the assumptions of the phylogenetic method [29]. The PHT is currently implemented with only parsimony, which assuming small number of actual sequence changes per site. Higher *P *values obtained by six-parameter weighting may indicates that, the six-parameter parsimony method fits the NT data better by accounting for the effect of multiple hits (as suggested in the saturation plot in Figure 1), thus reduces the incongruence in several pairwise comparisons.

### Phylogenetic analyses with NT data

**Figure 2:**
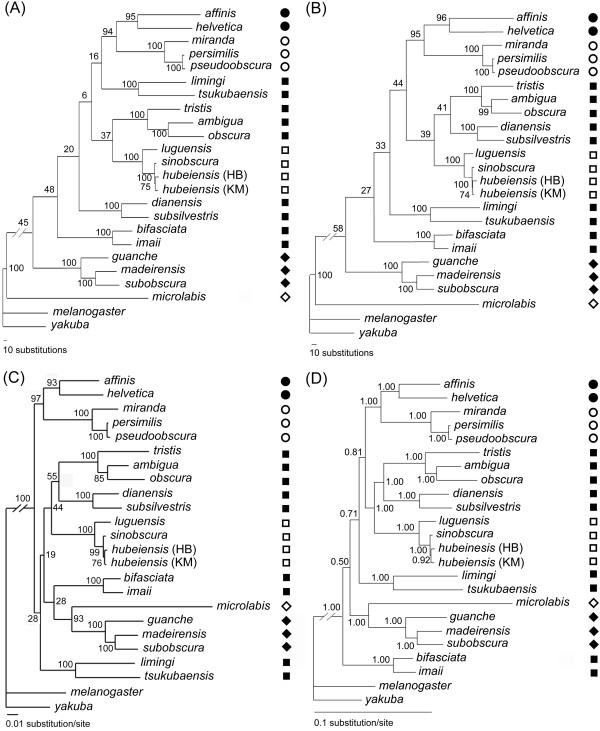
**Phylogenetic trees based on NT data**. (A) A single MP tree constructed with un-weighted scheme (tree length = 3233; CI = 0.4930; RI = 0.5581); (B) A single MP tree constructed with six-parameter weighting scheme (tree length = 2313.3101, CI = 0.5016, RI = 0.6059); (C) ML tree (-ln *L *= 20990.05); (C) Bayesian tree. Numbers besides nodes indicate bootstrap values in (A), (B) and (C), but posterior probabilities in (D). Symbols indicate the taxonomic assignment of species: *affinis *subgroup (●); *pseudoobscura *subgroup (○); *obscura *subgroup (■); *sinobscura *subgroup (□); *subobscura *subgroup (◆); *microlabis *subgroup (◇).

Results of analyses with NT data using the maximum parsimony (MP), maximum likelihood (ML) and Bayesian inference (BI) methods are shown in Figure 2. These analyses lend strong support to the monophyly of the *obscura *group, recover the same set of major lineages in this group, including the *affinis*, *pseudoobscura*, *subobscura *and *sinobscura *subgroups, the *D. ambigua *triad (*D. ambigua*, *D. obscura *and *D. tristis*), and the *D. dianensis*/*D. subsilvestris*, *D. bifasciata/D. imaii *and *D. limingi*/*D. tsukubaensis *lineages, each supported with high bootstrap value (BP) or posterior probability (PP). All the trees strongly support the sister relationship between the *affinis *and *pseudoobscura *subgroups (BP = 94–97; PP = 1.00). They completely agree with each other with respect to relationship within each of the major lineages. The branching orders within the *pseudoobscura *and *subobscura *subgroups are largely consistent with those of previous studies. The order within the *sinobscura *subgroup is also suggested by data of morphology [30] or interspecific reproductive isolation (Gao et al., unpublished data).

The MP tree deduced with the un-weighted method (Figures 2A; henceforth referred to as uwMP tree) clusters the *D. ambigua *triad with the *sinobscura *subgroup, while puts the *D. dianensis*/*D. subsilvestris *pair outside this cluster. However, the MP tree deduced with the six-parameter weighting method (Figure 2B; henceforth referred to as 6pMP tree), the ML tree (Figure 2C) and the Bayesian tree (Figure 2D) congruously suggest a cluster of the *D. ambigua *triad and the *D. dianensis*/*D. subsilvestris *pair. This cluster (henceforth referred to as *obscura *cluster) forms a larger cluster with the *sinobscura *subgroup (henceforth referred to as *obscura-sinobscura *cluster). However, the supports for these relationships are also low.

*D. microlabis*, as the single representative of the *microlabis *subgroup, is placed at the basal position in the uwMP and 6pMP trees. However, this species is clustered with the *subobscura *subgroup in the ML tree (Figure 2C, BP = 93) and Bayesian tree (Figure 2D; PP = 1.00). The uwMP, 6pMP and Bayesian trees suggest weakly (BP = 6–39; PP = 0.81) a close relationship between the *obscura-sinobscura *cluster and the New World clade. However, the ML tree clusters all the Old World species into a large group with weak support (BP = 28).

### Phylogenetic analyses with AA data set

**Figure 3:**
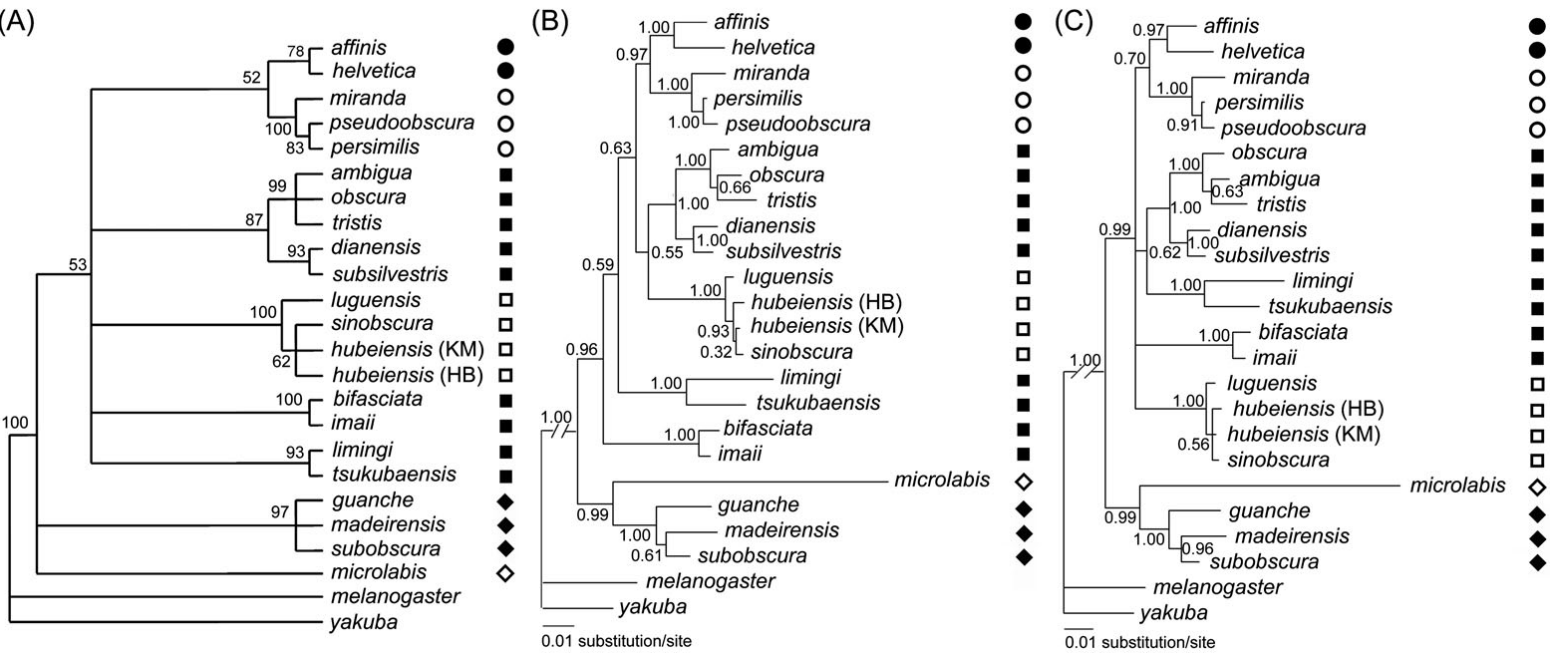
**Phylogenetic trees based on AA data set**. (A) Strict consensus of 17 equally parsimonious trees (tree length = 433, CI = 0.7367, RI = 0.7355); (B) Bayesian tree inferred with the Poisson model, with gamma-distributed rate variation across sites and a proportion of invariable sites; (C) Bayesian tree inferred with the GTR model, with gamma-distributed rate variation across sites and a proportion of invariable sites. Numbers besides nodes are bootstrap values of 1000 replicates in (A); those in (B) and (C) are posterior probabilities. Symbols are same as in Figure 2.

Figure 3 shows the results of phylogenetic analyses with AA data, including the strict consensus of 17 equally parsimonious MP trees (Figure 3A) and the Bayesian trees inferred with the Poisson model (Figure 3B) or the GTR (General time reversible) model (Figure 3C). All these trees recover the same set of major lineages as those trees of NT data, clearly indicating that the *microlabis *and *subobscura *subgroups are basal to the remainder ingroup species (BP = 53; PP = 0.96–0.99), all of which form a very large monophyletic group. The Bayesian analyses strongly indicate that *D. microlabis *forms a monophyletic group with the *subobscura *subgroup (PP = 0.99). All the analyses with AA data lend strong support to the monophyly of the *obscura *cluster (BP = 87; PP = 1.00), while the *obscura-sinobscura *cluster is only weakly suggested by the Bayesian tree inferred with the Poisson model.

The Bayesian trees of AA data recover the same relationships within each of the major lineages as those of the NT data, except for that they suggest a branching order of (*ambigua*, (*obscura*, *tristis*)) in the *D. ambigua *triad, and that the tree inferred with the Poisson model clusters the Kunming (KM) strain of *D. hubeiensis *with *D. sinobscura*, instead of its conspecific Hubei (HB) strain. However, all these relationships are very weakly supported.

### Divergence times in the *obscura *species group

**Figure 4:**
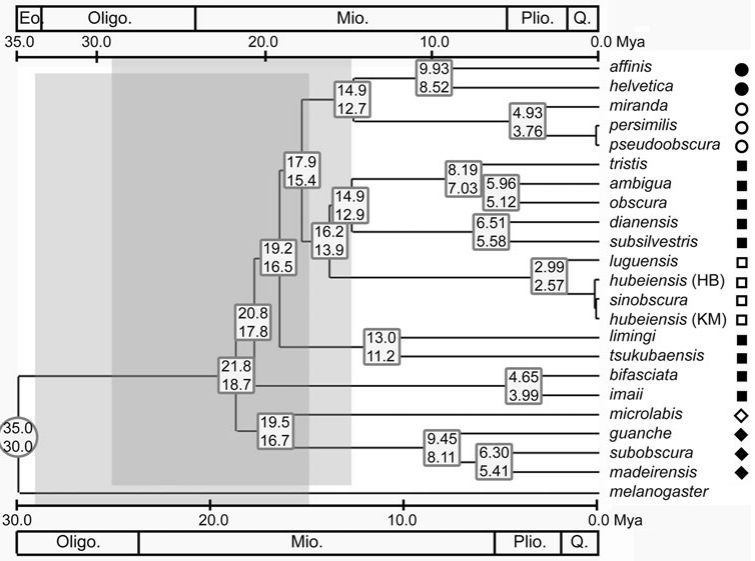
**Linearized ML tree deduced from concatenated nucleotide sequences of the 4 mitochondrial loci**. A time interval of 30–35 Mya for the *obscura-melanogaster *divergence [9] was used as calibration. Shaded areas (gray) indicate the time span of temperate forest developing in the Northern Hemisphere [50]. The numbers in the circle indicate the calibration points, and those in the panes indicate the estimated intervals of divergence times in light of the calibration points. For some very recent divergences, the estimates are not shown. Abbreviations: Eo. = Eocene; Oligo. = Oligocene; Mio = Miocene; Plio. = Pliocene; Q. = Quaternary. Symbols are same as in Figure 2.

The divergence times are estimated on a given tree – a ML tree constructed with the concatenated mitochondrial sequences. This tree is selected as a representative phylogenetic hypothesis of the *obscura *group based on the foregoing results. The results of time estimation are shown as a linearized form of the ML tree (Figure 4). In light of the upper bound (35 Mya) of the calibration time interval for the *obscura-melanogaster *divergence, the estimated time span for the major radiation of the *obscura *group is ~14.9–21.8 Mya, indicating that by the mid-Miocene time (~15 Mya), almost all the current major lineages of the *obscura *group had come into being. Even in light of the calibration point of 30 Mya, at least half of the major lineages are estimated to have originated by the mid-Miocene time. The splitting between the *D. ambigua *triad and the *D. dianensis*/*D. subsilvestris *pair (~14.9/12.9 Mya for upper/lower bounds) falls close to the major radiation of the *obscura *group, and the splitting between *D. limingi *and *D. tsukubaensis *(~13.0/11.2 Mya) appears to be rather old. The *sinobscura *subgroup began to diversify (~2.99/2.57 Mya) from about the mid-Pliocene time.

Our estimate for the origin of the *microlabis*-*subobscura *clade (~19.5/16.7 Mya) falls close to previous estimation based on mutation distance of 11 genes (17.7 ± 4.4 Mya; *D. pseudoobscura *vs. *D. subobscura*) [31]; the estimate for the splitting between the *affinis*-*pseudoobscura *clade and the *obscura*-*sinobscura *cluster (~17.9/15.4 Mya) is clearly older than the previous one based on *Adh *sequences (13.1 ± 1.74 Mya; *obscura *subgroup vs. *pseudoobscura *subgroup) [32], and the estimate for the *D. pseudoobscura*-*D. miranda *divergence (4.93/3.76 Mya) differs greatly from the estimate based on mutation distances (2.00 ± 0.6 Mya) [31]. This is mainly due to that 1) our estimates is not directly based on pairwise distances, but on a given tree with branch lengths; 2) we use different calibration point from those studies [31,32].

### Statistic test of the temporal pattern of evolutionary diversity

**Figure 5:**
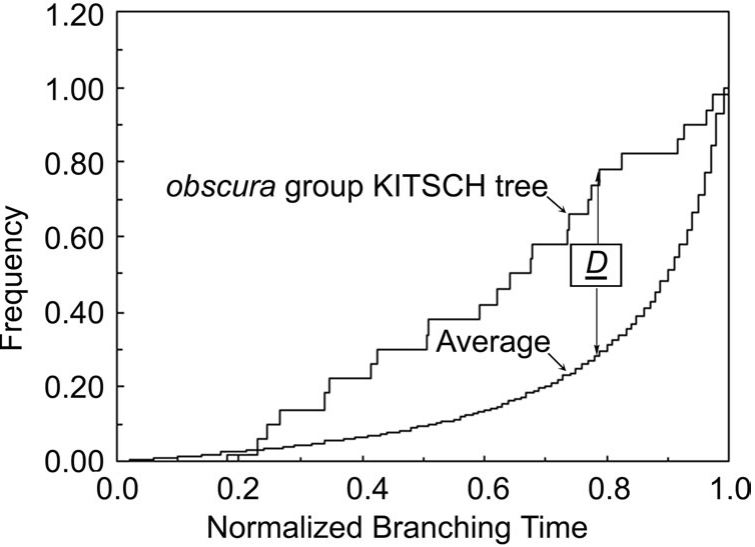
**CFD of normalized branching times for the KITSCH trees of 27 *obscura *group species**. The largest difference between empirical and expected CFDs is indicated.

To test the temporal pattern of the evolution of the *obscura *group, an empirical tree of 27 *obscura *group species (a KITSCH tree; not shown) is constructed under an assumption of molecular clock, therefore all the terminal taxa on this tree are shown as contemporaneous. The cumulative frequency distribution (CFD) of the branching times (after normalization) along this tree is plotted (Figure 5). An "expected" CFD for 27-taxa trees is provided as contrast. This expected CFD, available from Wollenberg et al [33], is the average of five hundred 27-taxa trees generated by computer simulation under null model of stochastic speciation/extinction. The comparison between the empirical and average CFDs with the Kolmogorov-Smirnov (K-S) test [34] results a K-S *D *value of 0.4975. The *P *value associated with this *D *value is very low (*P *< 0.0004, significant), therefore rejected the null hypothesis [33], indicating that the branching pattern in the KITSCH tree differs from the expected stochastic timing of speciation/extinction [33]. It is clearly seen that the CFD of the KITSCH tree begins to deflect upwards from the average CFD at a relative early stage of the branching process, suggesting an ancient cluster of branching events during the evolutionary history of the *obscura *group.

## Discussion

### Phylogenetic relationship in the *obscura *group

Phylogeny of the *obscura *species group is investigated with dense taxon-sampling from the Old World, especially the Oriental region, using both nucleotide and translated amino acid sequences of multiple loci. The results corroborate some previously well-recognized relationships, and shed some new light on the evolutionary history of the *obscura *group, especially the relationship among major lineages.

The MP trees of NT data suggest with low confidence that *D. microlabis *alone, as a long-branch taxon, represents the first branch in the *obscura *group. However, it was strongly suggested in the ML tree (Figure 2C) and Bayesian trees (Figures 2D, 3B, and 3C) that *D. microlabis *forms a monophyletic group with the *subobscura *subgroup. A suspicion arises whether the basal position of *D. microlabis *is true, or an artifact due to long-branch attraction (LBA) by those outgroups? As demonstrated by Anderson and Swofford [35], if this relationship is true, MP method is prone to positively recover it, thus seems to perform as good as, or even better than ML method. Otherwise, ML will outperform MP by recovering the true relationship. As shown by some studies with empirical data and/or computer simulation [35-37], model-based methods (ML and Bayesian methods) can be relatively robust against branch-length differences, even against model violation. Therefore, it is very likely that the basal relationship of *D. microlabis *alone in the MP trees is an artifact due to LBA, while the ML and Bayesian trees suggest the true relationship between the *microlabis *and *subobscura *subgroups.

The Bayesian trees with AA data (Figures 3B, 3C) support with high confidences a basal position of the *microlabis*-*subobscura *clade in the *obscura *group. This relationship is also suggested by previous cladistic analyses [13]. In some previous studies lacking any representative of the *microlabis *subgroup [10,12,38], the *subobscura *subgroup alone was placed as basal to the rest in the *obscura *group. Moreover, comparison of more than 48 morphological characters among *obscura *group species (except for the *microlabis *and *sinobscura *subgroups) suggests that *D. subobscura *(as the only representative of the *subobscura *subgroup) differs more from the other Eurasian species than the latter differ from each other [39].

The monophyly of the *obscura *cluster is also suggested by morphological data: in the *obscura *group, *D. dianensis*, *D. subsilvestris *and *D. obscura *are the only species characterized by pale spots on several abdominal tergites in female [30,40], and large, somewhat quadrate 10th sternite in male [30,41]. In the present study, the *obscura *cluster appears to be most closely related to the *sinobscura *subgroup, while the remainder species of the *obscura *subgroup, i.e., the *D. limingi*/*D. tsukubaensis *pair and *D. bifasciata*/*D. imaii *pair, appear to have diverged earlier. Based on these evidences, we propose here a revised notion of the *obscura *subgroup, i.e., the cluster of the five species *D. ambigua*, *D. obscura*, *D. tristis*, *D. dianensis *and *D. subsilvestris*.

Consistent with some previous study [27], the present study clusters the Palearctic *D. helvetica *with the Nearctic *D. affinis *with strong support, clearly indicating its adscription to the *affinis *subgroup. Morphologically, *D. helvetica *possesses some diagnostic characters pertained to the *affinis *subgroup, e.g., very small distal sex-comb, 6 rows of acrostichal setulae. Some morphological similarities between *D. helvetica *and *D. tolteca*, a member of the *affinis *subgroup, are also found [40]. Given the Old World Origin of the *obscura *group [42] and the monophyletic nature of the *affinis*-*pseudoobscura *clade, *D. helvetica *undoubtedly represents a refluence of the New World element back into the Old World.

It was demonstrated that Bayesian posterior probability can overestimate the true probability of node confidences if substitution model used for phylogenetic analysis is oversimplified [43], and/or if concatenated sequences data are used [44]. In the 6pMP and ML trees of NT data, the BP supports for the *obscura *cluster are relatively low, and so are the BP supports for the *obscura*-*sinobscura *cluster. However, the corresponding PP values in the Bayesian tree of NT data seem to be excessively high. Also in the analyses with AA data, a remarkable discrepancy between BP and PP is found for the large clade consisting of the *affinis*, *pseudoobscura*, *sinobscura*, and *obscura *subgroups. In our Bayesian analysis with NT data, partition-specific models are used. The Bayesian analyses of AA data with simple (Poisson) and comprehensive (GTR) models yield comparable PP supports for the above relationships. Therefore, the great discrepancy between BP and PP may be partially due to our using of concatenated sequences. On the other hand, it was shown that BP in ML analyses is generally a conservative estimate of statistical confidence [44], and that compared to the BP in ML analyses, BP in MP analyses shows lower correlation with Bayesian PP [45].

The effect of taxon sampling on phylogenetic accuracy has been addressed by a number of studies [46-49], most of which favor addition of taxa, especially for breaking up long branches to improve information about state of internal nodes and rate at individual sites [47]. In the present study, adding of a number of Old World taxa enables us to trace some additional, ancient branching events, resulted in some largely congruent basal relationship in the *obscura *group, e.g., the close relationship between the *D. ambigua *triad and the *D. dianensis/D. subsilvestris *pair, that between the *obscura *cluster and the *sinobscura *subgroup, and the sister relationship between the *microlabis *and *subobscura *subgroups. The *obscura *group is presently known for 41 described and at least 2 undescribed species. Future studies with denser taxon sampling and larger number of characters (especially for nuclear gene sequence characters) are desirable to fully resolve the basal relationship in this group.

### Temporal pattern of evolution

Throckmorton's [42] study with data of palegeography and fossil record has proposed that the founder of the *obscura *group arose and existed for short time before its expanding with the temperate forest. The temperate forest was proposed to began to spread in Northern Hemisphere with decreasing of temperature by about 10~15°C in Oligocene [50]. However, according to our time estimation (Figure 4), the Old World diversification of the *obscura *group began well after the origin of this group. By the mid-Miocene time, at least half of the current major lineages had come into being, indicating a more or less rapid major radiation of the *obscura *group. This is also suggested by the results of statistical test of temporal pattern (Figure 5), and reflected by the short internal branches in the phylogenetic trees (e.g., Figures 2C, 2D, 3B and 3C). On the other hand, obvious nucleotide substitution saturation (Figure 1) and base composition bias have been observed in the mitochondrial loci. All these results lend supports to the previous proposal [12] that either the rapid radiation, or the special evolutionary dynamics of the mtDNA in the *obscura *group may account for the phylogenetic predicament concerning the *obscura *group.

### Biogeography and history of adaptive radiation

Two major patterns during the evolution of the family Drosophilidae have been proposed by Throckmorton [42] based on morphological and biogeographical data: 1) the primary tropical disjunction involving species groups, subgenera and genera; and 2) the temperate-forest disjunction involving species subgroups and species, represented typically by the *obscura *species group. Due to lacking of data about drosophilid faunas from either the Afrotropical or the Oriental region, the disjunction pattern of the *obscura *group within the Old World was thought to be not clear [42]. However, the pattern is now much more clearly seen: there are about 14 species (3 of the *subobscura *subgroup, 4 of the *microlabis *subgroup, 1 of the *affinis *subgroup, 5 of the *obscura *subgroup, and 1 ungrouped) are restricted to or mainly distributed in Europe/the Afrotropical region; and at least 11 species (3 of the *sinobscura *subgroup, 6 of the *obscura *subgroup, and 2 undescribed) restricted to East/Southeast Asia. Among the Eurasian species of the *obscura *group, at least 9 are restricted or mainly distributed in the Oriental region, with the southmost records from the Mt. Kinabalu of Malaysia. This clearly indicates a thorough adaptation of the group into high-elevation temperate-like habitats in the Oriental region, a pattern parallel to those in the Afrotropical and Neotropical regions [8,51,52].

Our time estimation for the major radiation of the *obscura *group is overlapped largely to the hypothetical time span of the developing of temperate forest in Northern Hemisphere (mid-Oligocene to mid-Miocene) [50,53]. It was proposed based on biogeographical data that, from the mid-Tertiary times, the temperate drosophilid faunas developed and spread with the temperate forest, until the time of the temperate-forest disjunction in mid Miocene age [42]. It is very likely that the fragmentation of the temperate forest had enforced the Old World diversification, and that the gradual desertification of the Asia interior onset from the early Miocene epoch [54] played important role in enforcing the disjunction of the temperate forest and thus the east-west disjunction of the *obscura *group within the Old World.

The cooling of the climate in the Qinghai-Tibet area of South Asia resulted from the uplift of the Qinghai-Tibet Plateau in the Tertiary period was thought to provide favorable conditions for the Palearctic insect fauna to invade southwards [55]. It is reasonable to presume that changing of climate might have also facilitated the adaptation of the founders of the Oriental elements of the *obscura *group into South Asia. Probably the intensified uplift of the plateau in late Pliocene [55] has accelerated these elements (e.g., the *sinobscura *subgroup, initiated to diversify ~2.6–3.0 Mya) to spread around, giving rise to the current species in south China, India and Malaysia.

## Conclusion

In conclusion, our phylogenetic study suggests that, the *obscura *group began to diversify rapidly in the Old World before invaded into the New World. Among the Old World lineages, the *microlabis *and *subobscura *subgroups form a monophyletic group basal to the rest of the *obscura *group. Our results corroborate the finding by the previous studies that the traditional *obscura *group is paraphyletic, with some of its members (the *D. ambigua *triad plus the *D. dianensis*/*D. subsilvestris *pair) forming a monophyletic cluster, which appears to be most closely related to the *sinobscura *subgroup.

## Methods

### Samples, DNA extraction, PCR, cloning and sequencing

Samples of twenty-one species of the *D. obscura *group and one species of the *D. melanogaster *group (Table 2) were used for DNA sequencing. DNA was extracted from single fly by standard phenol-chloroform method. The PCR cycle program comprised an initial 2 min of predenaturation at 94°C, 35 cycles of amplification (50 s of denaturation at 94°C; 1 min of annealing at 55°C for *ND2 *and *COII*, 51.5°C for *Cyt b*, 52°C for *COI *and *Adh*, 60°C for *28S*; 1 min of extension at 72°C), and 5 min of sequence postextension at 72°C. The primers (all given left to right from 5' to 3' ends) for the PCR and sequencing of the *ND2*, *COI*, *COII*, *Cyt b *and *28S *genes were: nd2-1 ATATT TACAG CTTTG AAGG, and nd2-2 AAGCT ACTGG GTTCA TACC for the *ND2 *gene [56]; UEA5 AGTTC TAGCA GGAGC TATTA CTAT, and UEA8 AAAAA TGTTG AGGGA AAAAT GTTA for the *COI *gene [57]; coii-1 ATGGC AGATT AGTGC AATGG and coii-2 GTTTA AGAGA CCAGT ACTTG [13] for the *COII *gene; *Cyt b*-F TTATG GTTGA TTATT ACGAA, and *Cyt b*-R CAAAA CATAT GCTTA TTCAA for the *Cyt b *gene; 28S-H CCCGA AGTAT CCTGA ATCTT TCGCA TTG (designed by T. Katoh in Hokkaido University), and 28S-T TCTTA GTAGC GGCGA GCG [58] for the *28S *gene. PCR products were separated on 2.0% agarose gels, then excised from the gels and purified using Watson™ gel extraction mini kit (Watson Biotechnologies).

The *Adh *fragments of *D. hubeiensis *(KM), *D. luguensis*, *D. dianensis*, and *D. limingi *were amplified using the primers adh-e2+ CTGGAC TTCTG GGACA AGCG, and adh-e3- TAGAT GCCCG AGTCC CAGTG [27], and the PCR product was cloned into the PMD18-T Vector (TaKaRa), then transformed into *Escherichia coli *as host. Thereafter, the recombinant DNA was extracted then, and the *Adh *fragment was sequenced with the M13 universal primers AAGCT TGCAT GCCTG CAGGT CGACG and CGGTA CCCGG GGATC CTCTA GAGAT. After purifying of the product of sequence reaction, the sequences were determined using ABI 377 or ABI 3700 sequencer according to the protocol by manufacturer.

### Sequence aligning and characterization

The newly collected sequences were edited using the Editseq module of the DNAStar package [59]. For each of the *ND2*, *COI*, *COII*, *Cyt b*, *Adh *and *28S *gene fragment, homologous GenBank sequences were downloaded and aligned with newly determined sequences by the ClustalW method [60]. The intron region of the *Adh *gene was excluded from all analyses. The alignment was then adjusted by eye to make it conform to the codon assignments. Then the ends of a few *COI *and *28S *sequences were trimmed slightly, so as to make the majority of homologous sequences well overlapped. We use MEGA3 [61] to calculate base composition and ti/tv ratios in each data partitions. Detection of substitution saturation in mitochondrial and nuclear data partitions was performed by plotting the ratio ti/tv for sequence pairs versus corresponding number of whole substitutions with respect to codon positions (1st+2nd or 3rd), with the pairwise ratios and numbers of substitutions calculated in MEGA3 [61], and plots worked out with the Microsoft Excel program. Only ingroup species are included for saturation analysis.

### Pairwise partition homogeneity test (PHT) and phylogenetic analyses

Before the phylogenetic analyses, the NT data was subjected to pairwise PHT [62] between data partitions of different loci under either un-weighted or six-parameter weighting parsimony scheme [63,64] with PAUP* 4.0b10 [65], with heuristic search for 1000 replicates. Modeltest 3.6 [66] was used to estimate parameters of DNA substitution model for the six-parameter MP, ML and BI analyses.

Phylogenetic analyses with NT data set were performed using MP, ML and Bayesian inferring methods. The MP tree was constructed with either un-weighted or six-parameter weighting parsimony methods with heuristic search (initial trees obtained by 100 replicates random addition; branch swapping with TBR algorithm). For the six-parameter method, models specific to each locus were implemented in PAUP*4.0b10 [65], with each substitution classes was weighted based on its substitution rate (R_ij_, i.e., rate of transformation between nucleotide i and j) estimated with Modeltest3.6 [66]: w_ij _= -ln (R_ij_/∑R_i_). The weighting parameter stepmatrix for each locus was adjusted for satisfaction of triangle inequality in PAUP*4.0b10 [65]. To access the support level for each node on the MP trees, bootstrap (BP) [67] analyses were performed with 1000 replicates and heuristic search. The MP analysis with AA data was performed with similar strategy as that of the NT data set.

The ML analysis of NT data was performed using PAUP*4.0b10 [65], with parameters assigned as follows: base frequencies of A (respectively C, G and T) = 0.3089 (respectively 0.1385, 0.1319 and 0.4207); substitution rates of A-C (respectively A-G, A-T, C-G, C-T and G-T) = 2.0353 (respectively 13.2831, 6.7964, 5.9419, 33.5370 and 1.0000); proportion of invariable sites (*I*) = 0.5765; and gamma distribution shape parameter (*α*) = 0.8598.

Bayesian inferring was implemented in MrBayes3.1 [68]. The starting tree was randomly selected and four chains were run. For the analysis with NT data set, parameters are set as follows: "nst = 6" + "invgamma" applied to the character partition of mitochondrial genes, "nst = 6" + "gamma" to that of the *Adh *gene, and "nst = 2" + "gamma" to that of the *28S *gene. Bayesian analyses of AA data were performed with either the Poisson or the GTR models, with gamma-distributed rate variation across sites and a proportion of invariable sites. For all the Bayesian analyses, two independent runs were implemented in parallel, with the Markov chains been sampled every 100 cycles. The runs were stopped after 2,000,000 cycles of MCMC (Markov Chain Monte Carlo) for NT data, but 1,000,000 cycles for AA data, till the average deviation of split frequencies fall well below 0.01. For all the runs, 1,000 trees sampled at early phase of the chain (well before the end of this phase, the likelihood values stop to increase, and start to fluctuate within a stable range) were discarded, and the remainders were summarized to obtain a majority rule tree which showing all the compatible partitions.

### Estimates of divergence times in the *obscura *species group

Before estimating divergence times, relative-rate test using the program Phyltest2.0 [69] is conducted to examine constancy of sequence evolution between lineages in the *obscura *group. Since no time calibration point of fossil record or by geological dating is available for our estimation, we cite that used by Beckenbach et al. [9]: an interval of 30–35 Mya for the divergence between the *obscura *and *melanogaster *groups. The program r8s1.71 [70], which enables estimating divergence time in the absence of a molecular clock, is used to estimate the divergence times in the *obscura *species group. A ML tree constructed with mtDNA sequence data was used as the input tree file for time estimating. The model for the ML search is selected by Modeltest3.6 [66]: base frequencies are 0.3246, 0.1028, 0.1116 and 0.4610 for A, C, G and T, respectively; rates = 3.4175, 25.8349, 10.1514, 4.7640, 86.1667 and 1.0000 for A-C, A-G, A-T, C-G, C-T and G-T, respectively; *I *= 0.5629; *α *= 1.0481. A penalized likelihood (PL) [71] method is used for divergence time reconstructing, with a truncated Newton (TN) algorithm for finding optima of the objective functions. Cross-validation are checked over a range of smoothing values by set the parameters cvstart = 0, cvinc = 0.5 and cvnum = 10. Divergence time for all the nodes except for the root is estimated by rerunning of the input data with a selected smoothing parameter (= 316, which has the lowest cross-validation score) by checking of the cross-validation.

### Statistic test of the temporal pattern of evolutionary diversity

We perform a statistic test [33] of temporal pattern in the *obscura *group. To reduce the effect of incomplete sampling of extant taxa, GenBank sequences of some additional *obscura *group species (Table 3) are also used. Based on the aligned sequence of 27 *obscura *group species, a F84 distance data matrix is created, and a so-called KITSCH tree is constructed, using the program DNADIST and KITSCH, respectively, both packed in Phylip3.6 [72]. For distance estimating using DNADIST, shape parameters of the Gamma distribution (*α *= 1.0353) and base frequencies (A = 0.3132; C = 0.1401; G = 0.1320; T = 0.4147) are estimated in Modeltest3.6 [66], and the ratio of ti/tv (= 1.1) is calculated in MEGA3 [61]. The branching times for the resulted KITSCH tree were normalized between zero (the time of the first branching event) and one (the present), cumulative frequency distribution (CFD) [34] of the scaled branching times for all the nodes (*n *= 25) in the tree is plotted. The dissimilarity between the resulted empirical CFD and expected (i.e., average) CFD specific for same number of extant taxa was quantified using a Kolmogorov-Smirnov (K-S) goodness-of-fit statistic *D *[34]. The average CFD for specific number of extant species has been generated by Wallenberg et al. [33] by computer simulations under null model of stochastic lineage bifurcation and extinction. Therefore, we get the average CFD for 27 extant taxa by interpolated those for 25 and 30 taxa [33].

## Authors' contributions

JG participated in design of the study, acquired the data, analyzed the data, and drafted the manuscript; HW and TA participated in conception and design of the study, critically revised intellectual content of the manuscript; JP participated in analysis and interpretation of data; YZ conceived the study and participated in its design and data interpretation, and preparing the manuscript. All authors read and approved the final manuscript.
